# Prognostic Value of Dual-Time-Point [^18^F]FDG PET/CT for Predicting Distant Metastasis after Treatment in Patients with Non-Small Cell Lung Cancer

**DOI:** 10.3390/jpm12040592

**Published:** 2022-04-07

**Authors:** Sang Mi Lee, Jeong Won Lee, Ji-Hyun Lee, In Young Jo, Su Jin Jang

**Affiliations:** 1 Department of Nuclear Medicine, Soonchunhyang University Cheonan Hospital, Cheonan 31151, Korea; c91300@schmc.ac.kr; 2 Department of Nuclear Medicine, International St. Mary’s Hospital, Catholic Kwandong University, Incheon 22711, Korea; sads00@naver.com; 3 Department of Pulmonology, Allergy and Critical Care Medicine, CHA Bundang Medical Center, Seongnam 13496, Korea; plmjhlee@cha.ac.kr; 4 Department of Radiation Oncology, Soonchunhyang University Cheonan Hospital, Cheonan 31151, Korea; inyoung.jo@schmc.ac.kr; 5 Department of Nuclear Medicine, CHA Bundang Medical Center, CHA University, Seongnam 13496, Korea

**Keywords:** bone marrow, fluorodeoxyglucose F-18, non-small cell lung cancer, positron emission tomography, prognosis

## Abstract

This study aimed to evaluate the prognostic significance of 2-Deoxy-2-[^18^F]fluoro-D-glucose ([^18^F]FDG) uptake in the bone marrow (BM) and primary tumors on dual-time-point (DTP) PET/CT for predicting progression-free survival (PFS) and distant metastasis-free survival (DMFS) in patients with non-small cell lung cancer (NSCLC). We retrospectively analyzed DTP [^18^F]FDG PET/CT images from 211 patients with NSCLC. The maximum standardized uptake value (SUV) of primary lung cancer and mean [^18^F]FDG uptake of the BM (BM SUV) were measured from early and delayed PET/CT images, and the percent changes in these parameters (∆maximum SUV and ∆BM SUV) were calculated. On multivariate survival analysis, the maximum SUV and BM SUV on both early and delayed PET/CT scans were significantly associated with PFS, while the ∆maximum SUV and ∆BM SUV failed to show statistical significance. For DMFS, the ∆maximum SUV and ∆BM SUV were independent predictors along with the TNM stage. Distant progression was observed only in 1.3% of patients with low ∆maximum SUV and ∆BM SUV, whereas 28.2% of patients with high ∆maximum SUV and ∆BM SUV experienced distant progression. The ∆maximum SUV and ∆BM SUV on DTP [^18^F]FDG PET/CT were significant independent predictors for DMFS in patients with NSCLC.

## 1. Introduction

Non-small cell lung cancer (NSCLC) is the most common histopathological category of lung cancer and is notable for its poor clinical outcomes, with a 5-year overall survival rate between 10–20% [1]. With advancements in treatment modalities and strategies for NSCLC, a number of studies have investigated prognostic factors that could predict the risk of cancer progression and guide clinical decisions for managing patients with NSCLC [2]. Recently, several studies have shown that the inflammatory response of the host by the activation of immune cells plays an essential role in the development, growth, and metastasis of NSCLC cells [3,4,5]. In clinical observational studies, diverse serum inflammatory markers such as C-reactive protein (CRP), neutrophil-to-lymphocyte ratio (NLR), and platelet-to-lymphocyte ratio (PLR) turned out to be significantly related with clinical outcomes in patients with NSCLC, irrespective of the tumor stage and treatment modalities [6,7,8]. These results suggest that, in addition to tumor–intrinsic factors, the systemic inflammatory response of the host has a substantial prognostic value for predicting the progression of NSCLC [6,7,8]. 

In patients with NSCLC, 2-Deoxy-2-[^18^F]fluoro-D-glucose ([^18^F]FDG) positron emission tomography (PET)/computed tomography (CT) is a widely used diagnostic imaging examination for diagnosis, initial staging work-up, and prediction of clinical outcomes [9,10,11,12]. Until now, studies that have investigated the prognostic value of [^18^F]FDG PET/CT have utilized [^18^F]FDG uptake in the primary lung tumor as an imaging parameter, which reflects the metabolic activity of tumor lesions [11,13]. Along with tumor metabolic activity, the prognostic significance of the host inflammatory response has been evaluated using [^18^F]FDG PET/CT images by measuring [^18^F]FDG uptake in the bone marrow (BM) [11,12,14]. Increased [^18^F]FDG uptake in the BM is attributed to BM activation due to the immune response to cancer cells, and [^18^F]FDG uptake in the BM showed a significant association with survival and the risk of distant metastasis in patients with malignant diseases, suggesting the role of an imaging biomarker in reflecting the degree of host inflammatory response [11,12,14,15]. [^18^F]FDG uptake in malignant lesions increases upon delayed PET/CT imaging until 5 h after [^18^F]FDG injection, and the increment is related to the biological aggressiveness of tumors [16,17]. Based on these findings, several studies have utilized dual-time-point (DTP) [^18^F]FDG PET/CT scanning, which consists of early conventional imaging at 1 h and delayed imaging 2–3 h after [^18^F]FDG injection, and tried to assess the prognostic significance of [^18^F]FDG uptake in tumor lesions in patients with NSCLC [13,16,17]. However, in contrast to [^18^F]FDG uptake in tumor lesions, no studies have investigated the prognostic potential of [^18^F]FDG uptake in the BM on DTP [^18^F]FDG PET/CT.

Hence, in our study, we aimed to investigate the prognostic significance of [^18^F]FDG uptake in the BM and primary tumor on DTP PET/CT for predicting progression-free survival (PFS) and distant metastasis-free survival (DMFS) in patients with NSCLC.

## 2. Materials and Methods

### 2.1. Study Subjects

Retrospective review of the medical records was performed for 493 subjects who had DTP [^18^F]FDG PET/CT for the characterization of pulmonary nodules and/or mass lesions between March 2014 and August 2020 at CHA Bundang Medical Center. Of them, we finally recruited 211 patients (1) who were histopathologically diagnosed with NSCLC, (2) who showed no distant metastasis on staging examinations, and (3) who underwent surgical resection, radiotherapy, and/or chemotherapy for NSCLC after staging work-up. Patients (1) who had a history of another malignant disease, (2) who lacked blood test results including CRP and blood cell counts within a month before or after DTP [^18^F]FDG PET/CT, (3) who received supportive care without any curative or palliative treatment, and (4) who were lost to follow-up within 12 months after the treatment without recurrence were excluded from the study. Before the initial treatment, staging work-up comprising blood tests, bronchoscopy, contrast-enhanced chest CT, brain magnetic resonance imaging (MRI), and DTP [^18^F]FDG PET/CT was performed for the enrolled patients. The patients were treated with surgery, chemoradiotherapy, radiotherapy, or chemotherapy based on their clinical stage and condition. The mean interval between DTP PET/CT and the initial treatment was 11 ± 9 days. After treatment, the patients were followed-up with imaging studies at regular intervals of 3–6 months. Disease progression of NSCLC was diagnosed based on imaging examinations and/or histopathological confirmation. The patients with disease progression were categorized into two groups: those with locoregional disease progression and no distant metastatic lesions (locoregional progression group), and those with distant metastatic lesions irrespective of locoregional disease progression (distant progression group).

### 2.2. Hematologic Features

Blood laboratory data were obtained from blood tests at the staging work-up within a month before or after DTP [^18^F]FDG PET/CT. For all patients, the results of CRP, differential white blood cell (WBC) count, and platelet count were recorded. Using WBC and platelet counts, NLR and PLR were calculated. For each patient, four serum inflammatory markers (CRP, WBC count, NLR, and PLR) were used in the analysis

### 2.3. DTP [^18^F]FDG PET/CT

DTP [^18^F]FDG PET/CT was performed using a Biograph mCT (Siemens Healthineers, Germany). A dose of 5.18 MBq/kg [^18^F]FDG was intravenously injected after at least 6 h of fasting. The blood glucose level of all enrolled patients was less than 200 mg/dL at the time of the [^18^F]FDG injection. The early PET/CT scan was performed approximately 60 min after the [^18^F]FDG injection from the skull base to the mid-thigh and the delayed PET/CT scan was performed approximately 120 min after the injection for the chest region. A CT scan was performed at 100 kVp and 40 mA without contrast injection followed by a PET scan for 90 s in each bed position using the three-dimensional acquisition mode. PET images were reconstructed using an iterative ordered subset expectation maximization algorithm with attenuation correction.

### 2.4. PET/CT Image Analysis

Two nuclear medicine physicians, who were unaware of the clinicohistopathological characteristics and survival results of the patients, retrospectively evaluated all DTP [^18^F]FDG PET/CT images using OsiriX MD 10.11 software (Pixmeo, Geneva, Switzerland). For each patient, the maximum standardized uptake value (SUV) of the lung cancer and mean [^18^F]FDG uptake of the BM (BM SUV) were measured on both early and delayed PET/CT images. The volume of interest (VOI) was manually drawn on the primary tumor, and the maximum SUV of the tumor was measured. Subsequently, a spheroid-shaped VOI was placed over the vertebral body of five thoracic spines (T8–T12 spines), and an isocontour using a cut-off SUV of 75% of the maximum SUV of the VOI was automatically drawn within the VOI for each thoracic spine [18,19]. The mean SUV of voxels within the isocontour in the VOIs of the five selected thoracic spines was defined as the BM SUV. In patients who showed postoperative changes from previous spinal surgeries, compression fractures, and severe osteoarthritic changes in T8–T12 spines, other thoracic spines with no abnormal findings were selected for the measurement of the BM SUV [18,19]. Using the maximum SUV of the primary lung cancer and BM SUV on the early and delayed PET images, the percent change in both PET parameters (∆maximum SUV and ∆BM SUV) between the early and delayed PET scans was calculated as follows: (∆PET parameter) = ((parameter on delayed PET scan)–(parameter on early PET scan))/(parameter on early PET scan) × 100.

### 2.5. Statistical Analysis

We calculated Spearman rank correlation coefficients to evaluate the relationships between the BM parameters on DTP PET/CT images and serum inflammatory markers after assessing the normality of distribution. The Kruskal–Wallis test and post hoc comparisons with Dunn’s test were performed to assess the differences in DTP PET/CT parameters between the patient groups with no progression, locoregional progression, and distant progression. The prognostic significance of DTP [^18^F]FDG PET/CT parameters and clinicohistopathological factors for predicting PFS and DMFS was assessed using univariate and multivariate Cox proportional hazards regression analyses. For multivariate analysis, only variables that revealed a statistical significance on univariate survival analysis were included after adjusting for age and sex. Survival time was defined as the time from the day of initial treatment to the day of the detection of disease progression (distant progression for DMFS) or death from any cause. Patients with no disease progression were censored on the day of the last clinical follow-up. The optimal cut-off values of the PET/CT parameters were chosen by receiver operating characteristic (ROC) curve analysis, and the Kaplan–Meier method was used to estimate the survival curves of the PET/CT parameters to calculate the cumulative DMFS. The patients were classified into four subgroups based on the cut-off values of ∆maximum SUV and ∆BM SUV, and Fisher’s exact test was performed to assess the differences in distant metastasis rates between the patient subgroups. All data were statistically analyzed using MedCalc Statistical Software version 20.014 (MedCalc Software Ltd., Ostend, Belgium), and *p*-values of <0.05 were regarded statistically significant.

## 3. Results

### 3.1. Clinical Characteristics

**Figure 1:**
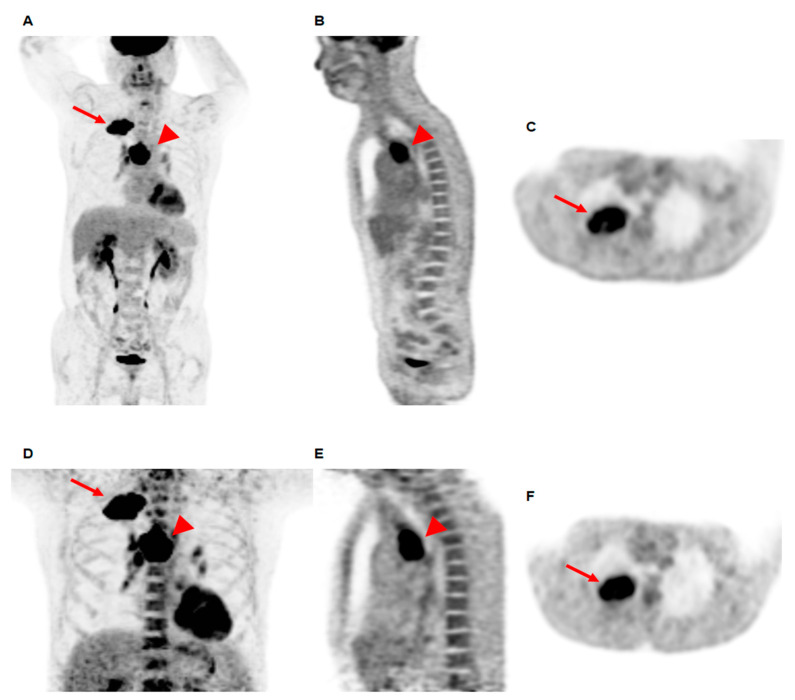
Maximum intensity projection (**A**), coronal (**B**), and transaxial (**C**) images of early PET/CT scan, and maximum intensity projection (**D**), coronal (**E**), and transaxial (**F**) images of delayed PET/CT scan of a 69-year-old man with squamous cell carcinoma. The primary lung cancer lesion at the right upper lobe (arrows on **A**,**C**,**D**,**F**) and metastatic lymphadenopathy at the right lower paratracheal area (arrowheads on **A**,**B**,**D**,**E**) show intensely increased [^18^F]FDG uptake. The maximum SUV of the primary lung cancer was 21.10 on early PET/CT and 28.57 on delayed PET/CT; thereby, ∆maximum SUV was 35.40. The BM SUV was 2.49 on early PET/CT and 3.65 on delayed PET/CT, resulting in a ∆BM SUV of 46.60. The patient was clinically diagnosed with a T3N2M0 stage and received concurrent chemoradiation therapy. At 11.2 months after the treatment, multiple distant metastatic lesions were newly detected and the cancer was determined to have progressed.

The clinicohistopathological characteristics and DTP [^18^F]FDG PET/CT parameters of the 211 enrolled patients with NSCLC are summarized in Table 1. In pairwise comparisons, the maximum SUV of the primary lung tumors and BM SUV on delayed PET/CT scans were significantly higher than those on early PET/CT scans (*p* < 0.001 for both). Of all patients, 15 (7.1%) and four (1.9%) showed decreased values of the maximum SUV of the primary tumor and BM SUV on delayed PET/CT images as compared to early PET/CT images, respectively. For the initial treatment, 143 patients were treated with surgical resection (67.8%) and, of them, 69 (48.3%, 69 out of 143) received adjuvant treatment after surgery. The duration of the median follow-up for the patients was 29.4 months with a range of 1.8–75.4 months. During follow-up, 94 patients (44.5%) experienced disease progression, with 75 and 19 in the locoregional and distant progression groups, respectively (Figure 1).

### 3.2. Correlation Analysis

The relationships between three BM parameters (BM SUV on early and delayed PET/CT scans and ∆BM SUV) and four serum inflammatory markers (CRP, WBC counts, NLR, and PLR) were evaluated (Table 2). The BM SUV on early and delayed PET/CT scans showed significant positive correlations with the CRP, WBC count, and NLR, whereas no significant correlation was observed with the PLR (*p* > 0.05). In contrast, the ∆BM SUV did not show significant correlations with any of the four serum inflammatory markers (*p* > 0.05). 

**Table 2:** Relationship between BM SUV parameters and serum inflammatory markers.

	BM SUV on Early PET/CT	BM SUV on Delayed PET/CT	∆BM SUV
CRP	r = 0.321 *p* < 0.001	r = 0.295 *p* < 0.001	r = 0.022 *p* = 0.755
WBC	r = 0.308 *p* < 0.001	r = 0.302 *p* < 0.001	r = 0.042 *p* = 0.546
NLR	r = 0.324 *p* < 0.001	r = 0.324 *p* < 0.001	r = 0.053 *p* = 0.441
PLR	r = 0.035 *p* = 0.614	r = 0.051 *p* = 0.466	r = 0.037 *p* = 0.594

**Figure 2:**
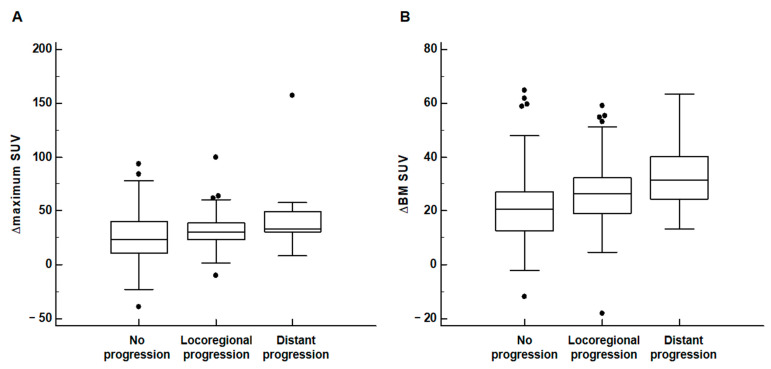
Distribution of the ∆maximum SUV of primary lung cancer (**A**) and ∆BM SUV (**B**) according to the disease progression pattern. (Black dot: an outside value which is larger than 75 percentile value plus 1.5 times the interquartile range).

BM SUV, mean standardized uptake value of bone marrow; CRP, C-reactive protein; CT, computed tomography NLR, neutrophil-to-lymphocyte ratio; PET, positron emission tomography; PLR, platelet-to-lymphocyte ratio; SUV, standardized uptake value; WBC, white blood cellThe relationship between the disease progression pattern and DTP PET/CT parameters was also assessed. The results of the Kruskal–Wallis test demonstrated that all DTP PET/CT parameters showed significant differences among patients with no progression, locoregional progression, and distant progression (*p* < 0.05; Table 3). Post hoc analysis showed that patients with locoregional and distant progression had significantly higher values of all PET/CT parameters than those with no progression (*p* < 0.05). On comparison between patients with locoregional and distant progression, there were no significant differences in the maximum SUV of the primary tumor and the BM SUV on early and delayed PET/CT (*p* > 0.10). Meanwhile, patients with distant progression tended to show higher ∆maximum SUV and ∆BM SUV values than those with locoregional progression with a borderline statistical significance (*p* = 0.092 for ∆maximum SUV and *p* = 0.064 for ∆BM SUV; Figure 2).

### 3.3. Survival Analysis

The prognostic significance of DTP [^18^F]FDG PET/CT parameters and clinicohistopathological factors for predicting PFS and DMFS on univariate and multivariate analyses is shown in Table 4 and Table 5. On univariate analysis, all six DTP PET/CT parameters showed significance in predicting PFS, along with age, sex, TNM stage, treatment, CRP level, WBC count, NLR, and PLR (*p* < 0.05; Table 4). For DMFS, the BM SUV on delayed PET/CT, ∆maximum SUV, and ∆BM SUV were significant prognostic factors, along with the TNM stage and NLR (*p* < 0.05; Table 4). Among the variables, those that showed statistical significance on univariate analysis were included on multivariate analysis. As the maximum SUV and BM SUV on early and delayed PET/CT scans were significantly correlated (*p* < 0.001, r = 0.905 for maximum SUV and *p* < 0.001, r = 0.847 for BM SUV), two separate multivariate models were used for predicting PFS: one with early PET/CT parameters and another with delayed PET/CT parameters. On multivariate analysis, the TNM stage, treatment, and maximum SUV of the primary tumor and BM SUV on both early and delayed PET/CT showed a significant association with PFS (*p* < 0.05), whereas both ∆maximum SUV and ∆BM SUV did not show prognostic significance (*p* > 0.05; Table 5). For DMFS, only the ∆maximum SUV (*p* = 0.008) and ∆BM SUV (*p* = 0.009) were independent predictors, along with the TNM stage (Table 5). An increase in the ∆maximum SUV and ∆BM SUV was associated with an increased risk of distant metastasis after treatment. 

**Figure 3:**
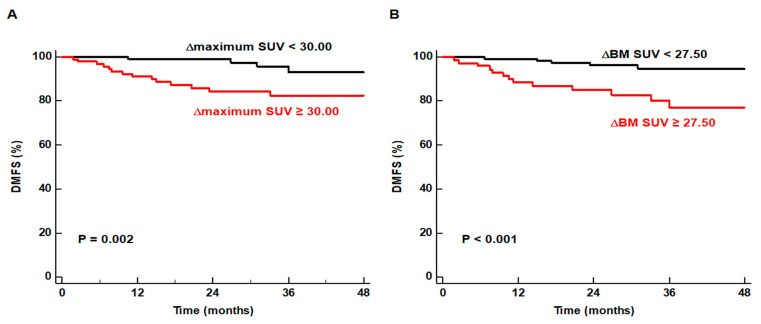
Kaplan–Meier curves for DMFS stratified according to the ∆maximum SUV of primary lung cancer (**A**) and ∆BM SUV (**B**).

For Kaplan–Meier analysis, the ∆maximum SUV and ∆BM SUV were categorized into two groups according to the specific cut-off values (30.00 for ∆maximum SUV and 27.50 for ∆BM SUV). The results of the Kaplan–Meier analysis demonstrated that patients with a high ∆maximum SUV and ∆BM SUV had significantly worse DMFS than those with low values (*p* = 0.002 for ∆maximum SUV and *p* < 0.001 for ∆BM SUV; Figure 3). 

A combination of the ∆maximum SUV and ∆BM SUV could further enhance the predictive value for the risk of distant metastasis. We categorized the patients into four subgroups according to the cut-off values of the ∆maximum SUV and ∆BM SUV and compared the distant metastasis rates between those four groups (Table 6). The patient subgroup with a high ∆maximum SUV and ∆BM SUV showed the highest distant metastasis rate (28.2%) after treatment among the four subgroups. This rate was significantly higher than that in the other three patient subgroups (subgroup with low ∆maximum SUV and ∆BM SUV: 1.3%, *p* < 0.001; subgroup with high ∆maximum SUV and low ∆BM SUV; 7.0%, *p* = 0.008; and subgroup with low ∆maximum SUV and high ∆BM SUV: 8.6%, *p* = 0.040). 

## 4. Discussion

In the present study, we investigated the prognostic significance of the BM SUV as well as the maximum SUV of NSCLC on DTP [^18^F]FDG PET/CT for predicting PFS and DMFS. For PFS, the maximum SUV and BM SUV on both early and delayed PET/CT were independent predictors, whereas ∆maximum SUV and ∆BM SUV did not show a significant association. In contrast, for DMFS, only the ∆maximum SUV and ∆BM SUV were independent prognostic factors, and the maximum SUV and BM SUV did not have any significant prognostic value. In the literature, this is the first study to demonstrate a significant relationship between changes in the maximum SUV of the primary tumor and BM SUV on DTP PET/CT with the risk of distant metastasis after treatment in patients with solid cancers. 

In previous studies, [^18^F]FDG uptake in the BM on PET/CT was found to not only correlate with the extent of systemic inflammatory response but also reflect the immune response to cancer [11,20,21,22]. In the correlation analysis, [^18^F]FDG uptake in the BM showed significant positive correlations with diverse serum inflammatory markers including NLR, CRP, WBC count, and PLR [11,14,15,23], which was also observed in our study. Furthermore, on survival analysis, [^18^F]FDG uptake in the BM was an independent prognostic factor for predicting PFS and overall survival in patients with head and neck, breast, lung, pancreatic, colorectal, and gynecologic cancers, consistently demonstrating worse survival in patients with a high [^18^F]FDG uptake in the BM [14,15,18,19,20,22,24]. For NSCLC, [^18^F]FDG uptake in the BM showed significant positive associations with clinical outcomes in patient cohorts treated with curative surgery, definite chemoradiotherapy, and chemotherapy [11,12,21]. In accordance with previous studies, our study also revealed that the BM SUVs on DTP PET/CT scans were significant prognostic factors for predicting PFS in multivariate survival analysis. In a previous study that enrolled patients with gynecologic cancers, those with a high [^18^F]FDG uptake in the BM demonstrated increased numbers of immunosuppressive immune cells, specifically myeloid-derived suppressor cells, and decreased numbers of antitumoral immune cells, such as cytotoxic T cells, in the peripheral blood and tumors [22]. Considering that the BM can regulate the balance between protumoral and antitumoral immunity, it could be assumed that increased [^18^F]FDG uptake of the BM in patients with cancer reflects enhanced immunosuppressive immune cell activities in the BM [25,26]. 

In addition to the significant association between PFS and BM SUV, the results of this study demonstrated that ∆BM SUV was a significant prognostic indicator for predicting DMFS. To date, only one study has investigated the prognostic value of changes in ∆BM SUV on DTP PET/CT [27]. This study enrolled 70 patients with diffuse large B-cell lymphoma and showed that patients with a ∆BM SUV of >45 had shorter overall survival than others [27]. Although the underlying mechanism for the relationship between ∆BM SUV and survival is yet to be elucidated, two possible explanations derived from the host and tumor factors could be suggested. Increased ∆BM SUV might represent the host BM conditions with highly activated immune cells, especially myeloid-derived suppressor cells and tumor-associated macrophages that promote epithelial–mesenchymal transition in NSCLC cells [28,29]. The aforementioned previous study also suggested that the increased ∆BM SUV could be attributable to the activation of the metabolic status of BM cells, which is induced by proinflammatory cytokines [27]. Another possible explanation is the presence of hidden metastatic cancer cells in the BM. Although patients who showed distant metastatic lesions on staging imaging examinations were excluded from the study, hidden microscopic metastasis might have been present in some of the enrolled patients, which would have affected the increase in ∆BM SUV and the risk of distant metastasis [30]. However, further studies are required to clarify the main mechanisms underlying this relationship. 

Along with the ∆BM SUV, the ∆maximum SUV of primary lung cancer is another imaging parameter of DTP PET/CT that showed a significant association with DMFS on multivariate survival analysis. In previous studies, the maximum SUV and ∆maximum SUV of tumors on DTP [^18^F]FDG PET/CT were found to be related to different aspects of cancer cell metabolism [31,32]. The maximum SUV of tumors on early PET/CT scans was mainly related to glucose transporter-1 expression in tumor cells, whereas the ∆maximum SUV had a close positive relationship with the expression ratio of hexokinase to glucose-6-phosphatase [31,32]. As the degree of hexokinase expression is related to the biological aggressiveness and proliferation rate of cancer cells, ∆maximum SUV is proposed as a promising imaging parameter that can reflect the malignant potential of cancer lesions [16,31,32,33]. However, clinical studies on patients with NSCLC regarding the prognostic value of the ∆maximum SUV have demonstrated inconsistent results. The ∆maximum SUV was shown to be an independent prognostic factor for predicting PFS and recurrence-free survival in several studies; however, other studies revealed that the ∆maximum SUV did not have any prognostic value for survival [13,16,33,34]. Another previous study that enrolled 57 patients with stage I NSCLC who had received stereotactic body radiation therapy demonstrated that the ∆maximum SUV was significantly associated with the risk of distant metastasis after radiotherapy, which is similar to the results of our study [35]. Furthermore, the study also revealed that the ∆maximum SUV tended to predict a lower risk of local recurrence and regional lymph node metastasis in NSCLC [35]. Considering these results, the ∆maximum SUV might have an inferior prognostic value compared with maximum SUV for predicting the risk of overall disease progression; however, it could be used as a potential indicator of distant progression of NSCLC. 

There is now ample evidence to suggest that the response of host immune cells to cancer cells, as well as the intrinsic features of cancer cells, is crucial for cancer progression and metastasis [36]. Based on this theory, recent studies have attempted to integrate imaging findings of primary tumors with imaging parameters that reflect the host’s immune response for a more precise prediction of clinical outcomes in patients with malignant diseases [14,20,37,38]. In previous studies of breast and pancreatic cancers, the risk of distant recurrence and death could be further stratified by merging BM findings with primary tumor imaging features [14,20]. Another study on patients with NSCLC also demonstrated that a model including imaging features of both primary tumors and BM more accurately predicted disease-free survival than clinical features alone [37]. Similarly, we could further stratify the risk of distant metastasis after treatment by combining the ∆maximum SUV of the primary tumor and the ∆BM SUV in patients with NSCLC. Our results showed that the distant metastasis rate in the patient subgroup with a high ∆maximum SUV and ∆BM SUV was up to 28.2%, which was significantly higher than that in all other patient subgroups. Although patients who experience distant progression have worse survival than those with locoregional progression, recent advances in NSCLC treatment are expected to lead to improved survival in patients with distant metastasis [39,40]. Therefore, more careful surveillance and intensive therapeutic intervention could be needed in patients with a high ∆maximum SUV and ∆BM SUV. Furthermore, when disease progression is suspected in these patients, whole-body imaging modalities such as [^18^F]FDG PET/CT should be preferentially considered for the possibility of distant progression. 

Our study has several limitations. First, as this was a retrospectively performed single-center study, which enrolled a limited number of patients, further studies are necessary to confirm our results. Second, TNM stage and treatment modalities of the enrolled patient population were heterogeneous, which might affect the results of the study. Third, because distant progression was diagnosed mainly by imaging studies, it is possible that the exact incidence of distant progression was underestimated. Fourth, previous studies have generally used 120 min after [^18^F]FDG injection as the imaging time for delayed PET/CT scans [13,16,17,34,35]; however, there is still no consensus on the best imaging time for the delayed PET/CT scan [32,33]. Finally, further investigations are required to identify the biological mechanisms underlying our results.

## 5. Conclusions

The changes in the maximum SUV of the primary tumor and BM SUV were significantly associated with the risk of distant progression after treatment in patients with NSCLC. Patients with high ∆maximum SUV and ∆BM SUV had worse DMFS than those with low values. The ∆maximum SUV and ∆BM SUV on DTP [^18^F]FDG PET/CT might provide prognostic information regarding distant progression in patients with NSCLC.

## Figures and Tables

**Table 1:** Patient characteristics (*n* = 211).

Characteristics		Number (%)	Median (Range)
Age (years)			67 (38–86)
Sex	Men	144 (68.2%)	
	Women	67 (31.8%)	
Smoking history	No	78 (37.0%)	
	Yes	133 (63.0%)	
Histopathology	Adenocarcinoma	135 (64.0%)	
	Squamous cell carcinoma	72 (34.1%)	
	Large cell carcinoma	3 (1.4%)	
	Adenosquamous carcinoma	1 (0.5%)	
T stage	T1–T2	163 (77.3%)	
	T3–T4	48 (22.7%)	
N stage	N0	130 (61.6%)	
	N1	28 (13.3%)	
	N2–N3	53 (25.1%)	
TNM stage	Stage I	103 (48.8%)	
	Stage II	40 (19.0%)	
	Stage III	68 (32.2%)	
Blood test	CRP (mg/dL)		0.29 (0.03–34.34)
	WBC (×10^9^ cells/L)		7.23 (2.97–35.60)
	NLR		2.09 (0.13–18.68)
	PLR		126.32 (11.93–2238.96)
Early PET/CT scan	Maximum SUV of primary tumor		12.20 (0.90–50.30)
	BM SUV		2.19 (1.28–3.66)
Delayed PET/CT scan	Maximum SUV of primary tumor		17.26 (0.55–64.02)
	BM SUV		2.66 (1.41–5.23)
∆PET parameter	∆Maximum SUV		27.92 (−38.89–157.29)
	∆BM SUV		23.12 (−17.98–119.85)
Treatment	Surgery	143 (67.8%)	
	Concurrent chemoradiotherapy	39 (18.5%)	
	Chemotherapy alone	17 (8.1%)	
	Radiotherapy alone	12 (5.7%)	

BM SUV, mean standardized uptake value of bone marrow; CRP, C-reactive protein; CT, computed tomography; NLR, neutrophil-to-lymphocyte ratio; PET, positron emission tomography; PLR, platelet-to-lymphocyte ratio; SUV, standardized uptake value; TNM, tumor, nodes, and metastases; WBC, white blood cell.

**Table 3:** Relationship of DTP PET/CT parameters with disease progression pattern.

PET/CT Parameters	No Progression (*n* = 117)	Locoregional Progression (*n* = 75)	Distant Progression (*n* = 19)	*p*-Value of the Kruskal–Wallis Test
Maximum SUV on early PET/CT	8.50 (3.44–13.75)	16.90 (13.10–21.80)	14.88 (9.18–19.40)	<0.001
BM SUV on early PET/CT	2.15 (1.87–2.34)	2.34 (2.01–2.68)	2.32 (1.91–2.91)	0.002
Maximum SUV on delayed PET/CT	11.61 (3.65–18.90)	21.90 (17.44–29.23)	19.60 (13.40–26.82)	<0.001
BM SUV on delayed PET/CT	2.53 (2.30–2.84)	2.96 (2.48–3.30)	3.12 (2.43–3.88)	<0.001
∆Maximum SUV	23.16 (10.92–39.98)	30.15 (23.32–38.63)	34.22 (30.89–49.35)	<0.001
∆BM SUV	20.68 (12.60–27.72)	26.04 (19.07–32.39)	31.45 (25.54–40.18)	<0.001

Data are presented as medians with interquartile range in parentheses. BM SUV, mean standardized uptake value of bone marrow; CT, computed tomography; DTP, dual-time-point; PET, positron emission tomography; SUV, standardized uptake value.

**Table 4:** Univariate analysis for PFS and DMFS.

Variables	PFS		DMFS	
	*p*-Value	Hazard Ratio (95% Confidence Interval)	*p*-Value	Hazard Ratio (95% Confidence Interval)
Age (1-year increase)	0.019	1.026 (1.004–1.049)	0.466	1.018 (0.971–1.067)
Sex (women vs. men)	0.003	2.111 (1.287–3.462)	0.209	2.031 (0.673–6.130)
Histopathology				
Adenocarcinoma vs. squamous cell carcinoma	0.420	0.841 (0.348–1.884)	0.377	0.646 (0.245–1.703)
Adenocarcinoma vs. large cell carcinoma and adenosquamous carcinoma	0.633	1.331 (0.411–4.308)	0.415	5.147 (0.652–24.867)
TNM stage				
Stage I vs. stage II	<0.001	3.634 (1.918–6.887)	0.173	2.226 (0.704–7.043)
Stage I vs. stage III	<0.001	12.717 (7.311–22.118)	0.012	4.791 (1.951–8.195)
Treatment				
Surgery vs. concurrent chemoradiotherapy	<0.001	4.803 (2.541–9.078)	0.531	1.614 (0.361–7.216)
Surgery vs. chemotherapy	<0.001	9.642 (5.925–15.688)	0.489	1.565 (0.440–5.575)
Surgery vs. radiotherapy	0.001	3.799 (1.764–8.182)	0.945	1.067 (0.140–8.169)
CRP (1.0 mg/dL increase)	<0.001	1.060 (1.024–1.097)	0.060	1.072 (0.997–1.153)
WBC (1.0 × 10^9^ cells/L increase)	0.020	1.055 (1.009–1.103)	0.126	1.098 (0.913–1.182)
NLR (1.0 increase)	<0.001	1.182 (1.111–1.258)	0.009	1.176 (1.056–1.309)
PLR (1.0 increase)	<0.001	1.001 (1.001–1.002)	0.641	1.000 (0.998–1.003)
Maximum SUV on early PET/CT (1.0 increase)	<0.001	1.057 (1.040–1.075)	0.118	1.035 (0.991–1.080)
BM SUV on early PET/CT (1.0 increase)	<0.001	2.662 (1.734–4.087)	0.063	2.604 (0.953–6.563)
Maximum SUV on delayed PET/CT (1.0 increase)	<0.001	1.047 (1.033–1.060)	0.068	1.030 (0.998–1.064)
BM SUV on delayed PET/CT (1.0 increase)	<0.001	1.975 (1.503–2.596)	0.011	2.563 (1.458–4.507)
∆Maximum SUV (1.0 increase)	0.013	1.009 (1.002–1.016)	0.009	1.017 (1.004–1.030)
∆BM SUV (1.0 increase)	0.029	1.012 (1.001–1.023)	0.006	1.025 (1.007–1.043)

BM SUV, mean standardized uptake value of bone marrow; CRP, C-reactive protein; CT, computed tomography; DMFS, distant metastasis-free survival; NLR, neutrophil-to-lymphocyte ratio; PET, positron emission tomography; PFS, progression-free survival; PLR, platelet-to-lymphocyte ratio; SUV, standardized uptake value; TNM, tumor, nodes, and metastases; WBC, white blood cell.

**Table 5:** Multivariate analysis for PFS and DMFS after adjustment of age and sex.

Variables	PFS with Early PET/CT Parameters		PFS with Delayed PET/CT Parameters		DMFS	
	*p*-Value	Hazard Ratio (95% CI)	*p*-Value	Hazard Ratio (95% CI)	*p*-Value	Hazard Ratio (95% CI)
TNM stage						
Stage II	0.048	2.091 (1.007–4.342)	0.045	2.107 (1.016–4.368)	0.224	
Stage III	<0.001	7.605 (4.089–14.106)	<0.001	7.592 (4.042–14.584)	0.009	4.541 (2.010–7.952)
Treatment						
Surgery vs. concurrent chemoradiotherapy	0.057		0.053		-	-
Surgery vs. chemotherapy	0.001	4.005 (2.024–7.924)	<0.001	4.059 (2.056–8.016)	-	-
Surgery vs. radiotherapy	0.014	3.092 (1.254–7.626)	0.016	3.057 (1.237–7.552)	-	-
CRP	0.084		0.060		-	-
WBC	0.240		0.201		-	-
NLR	0.417		0.451		0.134	
PLR	0.947		0.907		-	-
Maximum SUV on early PET/CT	0.015	1.033 (1.006–1.061)	-	-	-	-
BM SUV on early PET/CT	0.016	1.770 (1.114–2.8139)	-	-	-	-
Maximum SUV on delayed PET/CT	-	-	0.017	1.025 (1.005–1.046)	-	-
BM SUV on delayed PET/CT	-	-	0.018	1.556 (1.078–2.244)	0.201	
∆Maximum SUV	0.148		0.287		0.008	1.020 (1.005–1.035)
∆BM SUV	0.091		0.767		0.009	1.029 (1.010–1.052)

BM SUV, mean standardized uptake value of bone marrow; CRP, C-reactive protein; CT, computed tomography; DMFS, distant metastasis-free survival; NLR, neutrophil-to-lymphocyte ratio; PET, positron emission tomography; PFS, progression-free survival; PLR, platelet-to-lymphocyte ratio; SUV, standardized uptake value; TNM, tumor, nodes, and metastases.

**Table 6:** Distant metastasis rate during follow-up according to the combination of ∆maximum SUV of primary lung cancer and ∆BM SUV.

		∆BM SUV	
		<27.50	≥27.50
∆Maximum SUV of primary lung cancer	<30.00	1/80 (1.3%)	3/35 (8.6%)
	≥30.00	4/57 (7.0%)	11/39 (28.2%)

BM SUV, mean standardized uptake value of bone marrow; SUV, standardized uptake value.

## Data Availability

The datasets used and/or analyzed during the current study are available from the corresponding author on reasonable request.
